# Dynamic contrast-enhanced computed tomography to assess early activity of cetuximab in squamous cell carcinoma of the head and neck

**DOI:** 10.2478/raon-2014-0030

**Published:** 2015-03-03

**Authors:** Sandra Schmitz, Denis Rommel, Nicolas Michoux, Renaud Lhommel, François-Xavier Hanin, Thierry Duprez, Jean-Pascal Machiels

**Affiliations:** 1 Department of Medical Oncology and Head and Neck Surgery, Cliniques Universitaires Saint-Luc and Institut de Recherche Clinique et Expérimentale (Pole MIRO), Université Catholique de Louvain, Brussels, Belgium; 2 Department of Medical Imaging and Radiology, Cliniques Universitaires Saint-Luc and Institut de Recherche Clinique et Expérimentale (Pole IMAG), Université Catholique de Louvain, Brussels, Belgium; 3 Department of Nuclear Medicine, Cliniques Universitaires Saint-Luc, Université Catholique de Louvain, Brussels, Belgium

**Keywords:** cetuximab, head and neck cancer, perfusion, DCE-CT

## Abstract

**Background:**

Cetuximab, a monoclonal antibody targeting the Epidermal Growth Factor Receptor (EGFR), has demonstrated activity in various tumor types. Using dynamic contrast-enhanced computed tomography (DCE-CT), we investigated the early activity of cetuximab monotherapy in previously untreated patients with squamous cell carcinoma of the head and neck (SCCHN).

**Methods:**

Treatment-naïve patients with SCCHN received cetuximab for 2 weeks before curative surgery. Treatment activity was evaluated by DCE-CT at baseline and before surgery. Tumor vascular and interstitial characteristics were evaluated using the Brix two-compartment kinetic model. Modifications of the perfusion parameters (blood flow F_p_, extravascular space v_e_, vascular space v_p_, and transfer constant PS) were assessed between both time points. DCE data were compared to FDG-PET and histopathological examination obtained simultaneously. Plasmatic vascular markers were investigated at different time points.

**Results:**

Fourteen patients had evaluable DCE-CT parameters at both time points. A significant increase in the extravascular extracellular space v_e_ accessible to the tracer was observed but no significant differences were found for the other kinetic parameters (F_p_, v_p_ or PS). Significant correlations were found between DCE parameters and the other two modalities. Plasmatic VEGF, PDGF-BB and IL-8 decreased as early as 2 hours after cetuximab infusion.

**Conclusions:**

Early activity of cetuximab on tumor interstitial characteristics was detected by DCE-CT. Modifications of plasmatic vascular markers are not sufficient to confirm anti-angiogenic cetuximab activity in vivo. Further investigation is warranted to determine to what extent DCE-CT parameters are modified and to evaluate whether they are able to predict treatment outcome.

## Introduction

The Epidermal Growth Factor Receptor (EGFR) is overexpressed in up to 90% of all squamous cell carcinomas of the head and neck (SCCHN)^1^, and EGFR overexpression is linked with poor prognosis.^2^,^3^ The EGFR is a member of the HER tyrosine kinase receptor family composed of four different receptors (EGFR/c-erbB-1, c-erbB-2/HER-2/neu, c-erbB-3/HER-3, and c-erbB4/HER-4), all of which are transmembrane proteins with tyrosine kinase activity.^4^ The EGFR has an extracellular domain which provides a ligand-binding site. Upon ligand fixation, EGFR homodimerization or heterodimization with another HER receptor occurs leading to activation of the intracellular tyrosine kinase. This stimulates kinase signal transduction pathways involved in tumor proliferation, inhibition of apoptosis, angiogenesis and cell migration/invasion. Downstream signaling through the Ras/Raf/Mek/Erk pathway controls cell proliferation and cell cycle progression, while the phosphatidylinositol-3-kinase/protein kinase B (PI3K/Akt) pathway stimulates numerous antiapoptotic signals.

Cetuximab, a chimeric IgG1 monoclonal antibody (mAb) that specifically binds to the EGFR with high affinity, has been approved in combination with radiotherapy for locally advanced SCCHN and in combination with platinum based chemotherapy for recurrent and/or metastatic SCCHN.^5^–^7^ However the objective response rate in monotherapy remains low at between 10% and 13%.^8^ Postulated mechanisms of action of cetuximab include (i) downregulation of the EGFR and its downstream molecular signaling pathways by competing with EGFR natural ligands like EGF or TGF-alpha, (ii) antibody-dependent cell-mediated cytotoxicity (ADCC) through the activation of macrophages and natural killer cells, and (iii) inhibition of DNA double strand-break repair.^9^–^12^ Furthermore decreased secretion of vascular ligands like VEGF-A, IL-8 and FGF-2 has been described by other investigators(13,14), leading to the hypothesis that beside a direct effect on tumour cell proliferation, cetuximab could also induce anti-angiogenic effects and thereby influence indirectly tumor progression. Other investigators showed vascular normalization associated with increased vessel density and blood flow after anti-EGFR treatment in xenograft models.^15^,^16^

Dynamic Contrast-Enhanced Computed Tomography (DCE-CT) is now recognized as a useful non-invasive imaging tool to investigate vascular and interstitial tumor characteristics.^17^–^19^ In SCCHN, DCE-CT has been used to study blood volume, blood flow, permeability, tumor neoangiogenesis, and consecutive intratumoral arteriovenous shunts.^20^–^22^ These observations have been confirmed in several studies^22^–^25^ and were correlated with intratumoral microvessel density (MVD)^26^, and with pathological aggressiveness and angiogenic markers such as vascular endothelial growth factor.^27^–^29^ Several investigators have also used DCE-CT to try to establish predictive markers of treatment response after induction chemo-therapy or radio-chemotherapy in patients with SCCHN.^20^,^21^,^30^

We therefore implemented this technique in a pre-operative window opportunity study in SCCHN, in which cetuximab activity was investigated.^31^ Cetuximab monotherapy was administered for two weeks prior to surgery to treatment-naïve patients selected for primary surgical treatment. 2-[fluorine-18]-fluoro-2-deoxy-D-glucose positron emission tomography (^18^FDG-PET) and imaging, including DCE-CT, were performed at baseline and before surgery. It has been shown that ^18^FDG-PET and DCE-CT are complementary imaging techniques for surveillance assessment in patients with SCCHN and that their combination may improve tumor outcome prediction. 32 In this paper, we report on the effect of cetuximab monotherapy to modify the vascular and interstitial characteristics of SCCHN tumors as assessed by DCE-CT.

## Patients and methods

**FIGURE 1.**
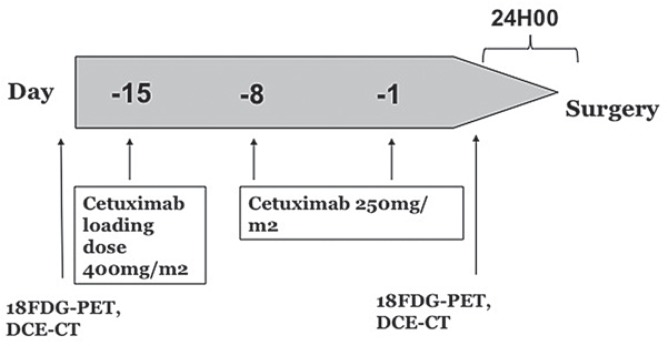
Schematic view of the study protocol.

Thirty-three treatment naïve patients with operable SCCHN were enrolled into a monocentric window pre-operative study between August 2008 and February 2011.^31^ In the first part of the study (N = 12 patients), safety of the concept was evaluated by progressive reduction of the delay between the last cetuximab administration and surgery. In the expansion part of the study (N = 20 patients), all patients were treated with a loading dose of 400 mg/m^2^ of cetuximab on day -15 before surgery followed by 250mg/m^2^ on days -8 and -1 before surgery (day 0) (Figure 1). Details of the eligibility criteria, ^18^FDG-PET responses of the whole group, biology and safety have been published.^31^ The clinical and translational parts of the study were approved by the Independent Ethics Committee and the Belgian Health Authorities and conducted in accordance with the Declaration of Helsinki (October 2000). Written informed consent was obtained for each patient. It was prospectively planned to perform translational research and patients gave their informed consent for repeated imaging.

### Perfusion imaging

DCE-CT scans were performed on the 20 patients treated in the expansion part of the study at two time points: before the first cetuximab infusion and strictly two hours after the third dose. Imaging used a 16-detector row CT scanner (Philips Medical Systems, Best, the Netherlands). Acquisition parameters were: tube voltage 90 kVp; reference tube current-time product 200 mAs, temporal resolution 1 s, total scan time 120 s, number of slices 4, in-plane spatial resolution 0.68×0.68 mm, slice thickness 6 mm, image matrix size 512×512. Raw data were reconstructed by using a FBP algorithm. PET-CT fusion was used to locate the lesions on CT images.

**FIGURE 2.**
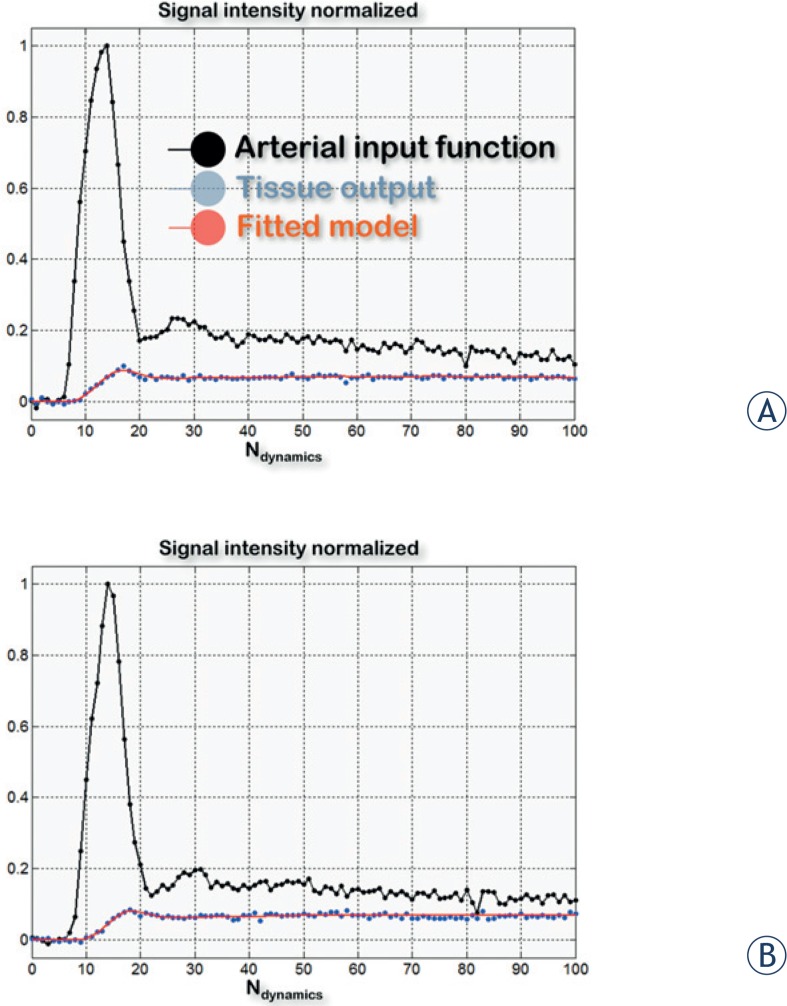
Example of time intensity curves derived from the internal carotid and tumoral lesion before **(A)** and during treatment with cetuximab **(B)** in a patient with a squamous cell carcinoma of the head and neck. The quality of the fits of TICs using the Brix two-compartment kinetic model was found to be good (estimation of the root mean squared error of the regression averaged on the 14 normalized fits: ε^pre-treatment^ = 5%, ε^during-treatment^ = 6%).

The software Image J (processing program developed by the National Institutes of Health, http://rsbweb.nih.gov/ij/) was used to segment the regions of interest (ROI). An expert head and neck radiologist manually drew the ROI which included the internal carotid (for the arterial input function calculation) and the whole tumor (Figure 2).

Time intensity curves (TICs) were analyzed according to the Brix two-compartment kinetic model (Figure 2).^33^ This model relies on four parameters: blood flow F_p_ (mL.s^−1^.g^−1^); fraction of extravascular extracellular space accessible to the contrast agent v_e_ (%); fraction of vascular space v_p_ (%); and the transfer constant PS (mL.s^−1^.g^−1^), which depends on the permeability and surface area for transendothelial exchanges of the capillary wall. An additional free parameter τ (s), to account for the contrast agent bolus arrival time in the tissues of interest, was included. As a result, the final expression of the kinetic model was:

[1] $${\text{C}}_{\text{tissue}}\;\left(\text{t}\right)={\text{F}}_{\text{p}}.{\text{C}}_{\text{plasma}}\left(\text{t}-\tau \right)⊗{\text{R}}_{\text{tissue}}\left(\text{t}\right)$$

where C_plasma_(t-τ) is the TIC in the feeding artery and R_tissue_(t) is the impulse response function of the tissue taken the form:

[2] $$\begin{array}{l} {\text{R}}_{\text{tissue}}\left(\text{t}\right)=\text{A}.{\text{e}}^{-\alpha \text{t}}+\left(1-\text{A}\right).{\text{e}}^{-\beta \text{t}}\\ {\text{V}}_{\text{p}}={\text{F}}_{\text{p}}/\left[\text{A}.\left(\alpha -\beta \right)+\beta \right]\\ \text{PS}=\left[\alpha +\beta -\alpha \beta /\left(\text{A}.\left(\alpha -\beta \right)+\beta \right)\right].{\text{v}}_{\text{p}}\\ {\text{V}}_{\text{e}}=\text{PS}/\left[\alpha \beta \text{/}\left(\text{A}.\left(\alpha -\beta \right)+\beta \right)\right] \end{array}$$

The model was fitted to the TICs using a trust region algorithm.^34^ Multiple start values for the free parameters were explored in an attempt to find the solution corresponding to the true global minimum of the error function. All parameters were constrained to be positive. The sum (v_e_ + v_p_) was constrained to be less than 1. No upper boundary was imposed on F_p_ or PS. To obtain the coefficients F_p_ and PS in mL.min^−1^.100g^−1^, the coefficients expressed in mL.s^−1^.g^−1^ were multiplied by 60 s min^−1^ and by 100. Cetuximab-induced changes in the pharmacokinetic parameters F_p_, v_e_, v_p_ and PS were then investigated.

### 18FDG-PET imaging

All patients underwent two ^18^FDG-PET/CT assessments on a Philips 16-slice GEMINI TF camera (Philips Healthcare, The Nederlands): a baseline PET at the time of inclusion and an evaluation ^18^FDG-PET aimed at estimating the residual tumor metabolic activity at the end of the cetuximab regimen (performed strictly 2 hours after the final infusion of cetuximab and one day before surgery).

As a surrogate of tumor metabolic activity, the maximal standardized FDG uptake value (SUV_max_) was recorded at each time point within the entire tumor volume using 3D volumes of interest (VOIs) and calculated using the following formula:

[3] $${\text{SUV}}_{\text{max}}=\text{maximal}\;\text{pixel}\;\text{value}\;*\text{weight}/\text{corrected}\;\text{injected}\;\text{dose}*1000$$

with SUV_max_ in g.mL^−1^, maximal pixel value in Bq.mL^−1^, weight in kg, and the dose in Bq. To reflect the modification in tumor metabolism induced by the treatment between two PET studies, the parameter ΔSUV_max_ (in %) was defined using the following formula:

[4] $$\Delta {\text{SUV}}_{\text{max}}=[\left({{\text{SUV}}_{\text{max}}}^{\text{E}-\text{PET}}-{{\text{SUV}}_{\text{max}}}^{\text{B}-\text{PET}}\right)/\;{{\text{SUV}}_{\text{max}}}^{\text{B}-\text{PET}}]*100$$

Based on published EORTC criteria for solid tumor evaluation with PET^35^, tumor metabolism was considered to be progressive (non responding to treatment) if ΔSUV_max_ was > 25% between the two PET studies; stable for ΔSUV_max_ between −25% and +25%; partially responding for ΔSUV_max_ ≤ −25%, and in complete metabolic response in case of non residual uptake. The PET-CTs were centrally reviewed by the same person.

### Tumor cellularity

Residual tumor cellularity was determined on hematoxylin and eosin-stained (HE) slides including the whole tumor as previously described.^31^ Stained slides were digitized by a slide scanner (Mirax Scan; Zeiss, Jena, Germany). The surface composed of tumor cells only and the surface of the whole tumor were manually drawn by the same investigator (SS) who was blinded to the DCE-CT and ^18^FDG-PET results. Tumor cellularity (expressed as a %) was the surface occupied by tumor cells divided by the surface of the whole tumor that included tumor cells, inflammatory cells, normal interstitial tissue and areas with morphologic signs of therapy-induced regression such as fibrosis and scaring.

### Plasma analyses

Plasma samples (3 ml) were collected at several time points: (T1) at baseline before any cetuximab infusion; (T2a) before the last dose of cetuximab (day -1 before surgery); (T2b) 2 hours after the last dose of cetuximab; (T3) before surgery after intubation; and (T4) 5 weeks after surgery.

Vascular endothelial growth factor (VEGF), fibroblast growth factor (FGF)-basic, platelet derived growth factor (PDGF)-BB and interleukin (IL)-8 plasma levels were quantified using the Bio-Rad multiplex bead immunoassay (Luminex). The assay was performed in a 96 well plate format and analysed with the Luminex200 instrument (BIORAD), which monitors the spectral properties of the capture beads while simultaneously measuring the quantity of associated fluorophore.

### Statistical analysis

Numerical variables were expressed as mean ± standard deviation. DCE-CT parameters, ^18^FDG-PET parameters, tumor cellularity and Luminex results were compared before and during treatment using the non-parametric Wilcoxon rank-sum test. A non-parametric test was chosen as the normality of the data distribution was not verified (on the basis of the D’agostino-Pearson test). Correlations between parameters were assessed based on Spearman’s rank coefficient. All calculations were done with Matlab (Matlab R2011b, MathWorks, Natick, MA, USA). A *p*-value < 0.05 was regarded as statistically significant for all tests cited above.

## Results

### Study population

Twenty patients were included in the expansion part and treated with three doses of cetuximab. DCE-CT images were spatially registered with a rigid transformation in order to compensate for patient motions.^36^ However, in four patients out of 20, displacement or deformation of the oral cavity during the perfusion sequence was too severe to be corrected. In two additional patients, dental artifacts prevented from measuring the first pass of the contrast agent in the ROI with a high signal to noise ratio. As a result, 14 patients out of the original 20 qualified at both imaging time points for a quantitative assessment of the tumoral perfusion. Eleven and three patients had SCC of the oral cavity and larynx, respectively.

### Anatomic imaging

Nine out of the 14 patients had measurable lesions according to response evaluation criteria in solid tumor (RECIST) version 1.1. The largest diameter of the tumor decreased by more than 30% in only one patient. Other patients had stable disease (SD) according to RECIST criteria.

### Functional imaging and tumor cellularity

Quantitative imaging parameters are summarized in Table 1. A significant difference between pre-treatment and during cetuximab treatment was observed for the extravascular extracellular space v_e_ (*p* = 0.0085). Other kinetic parameters were not found to differ significantly (F_p_: *p* = 0.5416; v_p_: *p* = 0.2958; PS: *p* = 0.5830).

Among the 14 patients, all except one had a partial response (PR) according to the ^18^FDG-PET EORTC guidelines. Only one patient had an increase in SUV_max_ between the two ^18^FDG-PET scans (ΔSUV_max_ + 15%). For the other patients, ΔSUV_max_ was between −25% and −50% for 7 patients and below −50% for 6 patients.

We have previously shown that cetuximab decreased tumor cellularity in resected specimens compared to untreated patients.^31^ Analysis of tumor cellularity in HE slides of resected tumor specimens showed a median cellularity of 43% [range: 22% – 77%]. Tumor cellularity was more pronounced for the 3 larynx carcinomas [range: 54% – 77%] than in the 11 tongue carcinomas [range: 22% – 51%]. Ten out of the 14 patients (71%) had a tumor cellularity < 50%.

A significant though moderate inverse correlation between the extravascular extracellular space v_e_ and SUV_max_ (ϱ = −0.40, *p* = 0.03) was observed. No other correlation was observed between DCE-CT parameters and ^18^FDG-PET parameters and tumor cellularity, or between the delta values of the respective parameters. A significantly strong correlation was found between cellularity and ΔSUV_max_ (ϱ = 0.84, *p* = 0.0003) in these 14 patients.

### Plasma analysis

**FIGURE 3.**
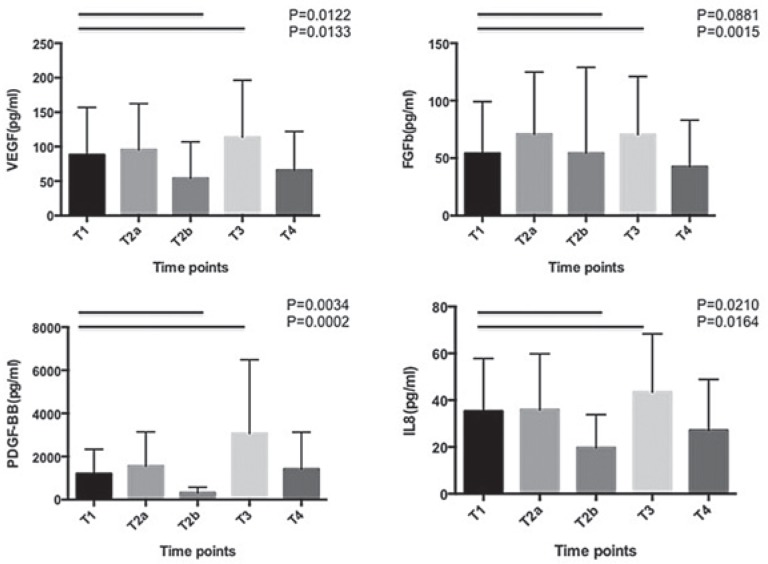
Modifications of VEGF, FGF-basic, IL-8 and PDGF-BB plasmatic levels at different time points. T1: baseline sample, T2a: after 2 doses of cetuximab and before the third dose of cetuximab; T2b: 2 hours after the third dose of cetuximab and just before ^18^FDG-PET (24 hours before surgery); T3: at induction of anesthesia, before incision; T4: 5 weeks after surgery. (Representation of the mean and SD for each time point, n=14 pts)

VEGF, FGF-basic, PDGF-BB and IL-8 plasmatic levels decreased as early as 2 hours (T2b) after cetuximab perfusion. At the moment of surgery, the plasmatic levels of all these ligands were significantly upregulate (Figure 3).

## Discussion

The main purpose of this study was to investigate, using DCE-CT, the ability of cetuximab monotherapy to modify a tumor’s vascular and interstitial characteristics. Cetuximab is known to block EGFR-dependent intracellular downstream molecular pathways.

In our model, a significant increase in the extravascular extracellular space v_e_ between pretreatment and during treatment was observed. In addition to the modification of v_e_, a ^18^FDG-PET partial response according to EORTC criteria and tumor cellularity inferior to 50% in the resected specimens were observed in 90% and 71% of the 14 reported patients, respectively.

**FIGURE 4.**
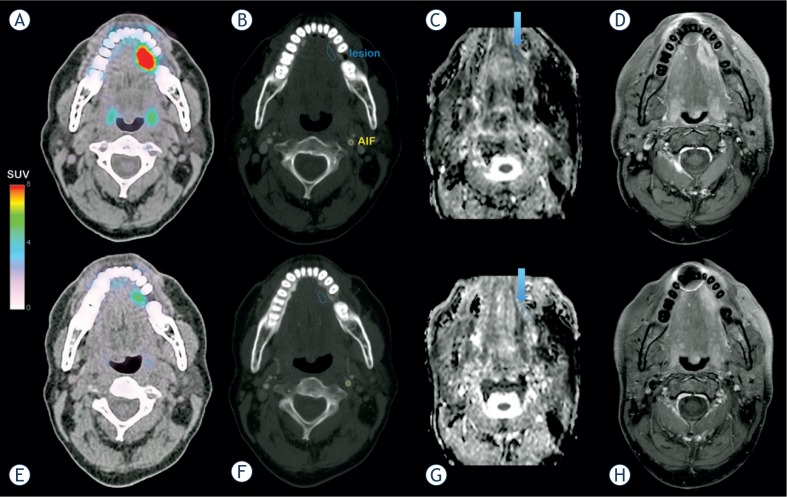
A patient with squamous cell carcinoma of the head and neck. Before cetuximab treatment: **(A)** PET/CT fusion imaging showing the anatomical location of FDG uptake. **(B)** CT imaging with the regions of interest used for kinetic analysis. **(C)** ADC mapping derived from DW-MRI with a measured ADC^lesion^ = 1009 mm^2^.s^−1^ ± 123 mm^2^.s^−1^. **(D)** Spin Echo T1-weighted MR imaging with fast suppression technique (SPIR) after gadolinium injection (lesion long axis: 2.88 cm, lesion short axis: 1.52 cm). MRI examination was performed 6 days after PET/CT. During treatment with cetuximab: **(E)** PET/CT fusion showing imaging of the decrease of FDG uptake. **(F)** CT imaging with the regions of interest used for kinetic analysis. **(G)** ADC mapping derived from DW-MRI with an increased ADC^lesion^ = 1325 mm^2^.s^−1^ ± 192 mm^2^.s^−1^. **(H)** Spin Echo T1-weighted MR imaging with fast suppression technique (SPIR) after gadolinium injection (lesion long axis: 2.72 cm, lesion short axis: 1.12 cm). MRI and CT examinations were performed the same day.

Modifications on blood flow F_p_, vascular space v_p_ or transendothelial exchanges PS could not be demonstrated. Since most of our patients did not experience tumor shrinkage, our results suggest that cetuximab induces modifications in tumor composition reflected by reduced metabolic tumor activity (ΔSUV_max_) and increased peritumoral space, as shown by an increase in v_e_ and a decrease in tumor cellularity. These modifications are consistent with our previous report based on the apparent diffusion coefficient (ADC) parameter derived from diffusion-weighted magnetic resonance imaging (DW-MRI).^31^ Here, a measure of the mobility of water molecules in tissues, as influenced by physiological barriers (endothelium, cell membrane) and obstacles (constituents of the extracellular matrix, intracellular organelles), showed an increase in three out of four patients, from a mean ADC^lesion^ = 1065 mm^2^.s^−1^ ± 139 mm^2^.s^−1^ to a mean ADC^lesion^ = 1226 mm^2^.s^−1^ ± 209 mm^2^.s^−1^ after cetuximab.^23^ An example of ^18^FDG-PET, DCE-CT and DW-MRI for one patient before and after treatment is illustrated in Figure 4.

Although less investigated and characterized, anti-angiogenic properties of cetuximab have also been described *in vitro*. Luwor *et al.* found a reduction of hypoxia-inductible factor 1-α (HIF-1α) and transcriptional inhibition of vascular endothelial growth factor (VEGF) expression in response to cetuximab treatment.^37^ According to previous reports^13^,^14^,^38^, we also observed decreased secretion of VEGF, FGF-2 and IL-8 supporting anti-angiogeneic effects of cetuximab. These modifications occurred as early as 2 hours after cetuximab administration. However, at the moment of surgery, we noticed a significantly increase of these proteins probably induced by the operative stress.^39^,^40^ Furthermore, vascular normalization and normalized tumor oxygenation was observed in xeno-graft models treated by EGFR-TKIs.^15^,^16^ These data were not confirmed by the present *in vivo* study which failed to demonstrate a significant change in vascularization parameters recorded by DCE except for the v_e_ which could be explained by the cetuximab-induced cytoreduction, as shown by the decreased tumour cellularity and metabolic imaging.

The main limitation of our study was the low number of patients assessed by DCE-CT. Statistical considerations to determine the number of patients to be included in this study were based on ^18^FDG-PET responses^31^ - the DCE-CT analyses were exploratory and hypotheses generating. However, the number of patients was sufficient to observe differences in tumor metabolism and extravascular extracellular space composition induced by the therapy but probably insufficient to detect slight modifications in tumor vascularization.

## Conclusions

To our knowledge, this is the first article to study the variation of DCE-CT and ^18^FDG-PET parameters in patients with SCCHN treated with cetuximab monotherapy. A significant increase in the extravascular extracellular space v_e_, as well as a decrease in tumor metabolism by ^18^FDG-PET, was observed. Further investigation in larger cohorts of patients is warranted to assess the extent of change in perfusion measurements prior to and during treatment. Data from such studies could ideally translate into values that could predict treatment efficacy and provide information on the potential effects on tumor blood flow and vascular volume. Since only a minority of patients benefit from targeted therapies, identification of early imaging markers to predict treatment outcome would be of great value to the oncology community.

## Figures and Tables

**TABLE 1.** Quantitative imaging parameters from DCE-CT and ^18^FDG-PET before and during treatment with cetuximab in 14 patients with squamous cell carcinoma of the head and neck

**Patient**	**F**_p_	**v**_e_*	**v**_p_	**PS**	SUV*	**Cellularity**	**Tumor**

	**(mL.min**^−1^**.100g**^−1^**)**	**(%)**	**(%)**	**(mL.min**^−1^**.100g**^−1^**)**		**(%)**	**localization, staging**

**Pretreatment**							
**1**	313.7	30.3	8.1	53.8	14.3	-	L, T2N0
**2**	378.6	39.2	12.0	59.7	16.8	-	OC, T2N0
**3**	157.2	15.4	16.0	37.7	10.6	-	OC, T2N0
**4**	366.5	60.5	13.7	81.9	11.4	-	OC, T1N0
**5**	73.9	41.8	15.3	28.6	9.9	-	OC, T1N0
**6**	136.1	30.9	11.1	69.2	13.4	-	OC,T4N0
**7**	278.0	36.4	15.5	47.3	8.8	-	L, T2N0
**8**	116.9	39.5	13.6	42.0	8.3	-	OC, T2N1
**9**	93.7	41.9	13.0	82.9	16.2	-	OC, T2N1
**10**	163.2	21.9	9.5	37.2	12.3	-	L, T2N0
**11**	72.7	29.1	9.2	38.1	7.4	-	OC, T2N0
**12**	286.6	49.8	5.6	40.9	28.9	-	OC, T3N0
13^***^	128.0	37.0	10.8	44.6	14.8	-	OC, T2N0
**14**	222.8	21.1	18.7	36.2	18.9	-	OC, T2N2b
**m ± SD**	**199 ± 107**	**35 ± 12**	**12 ± 3.5**	**50 ± 17**	**14 ± 5.6**	**-**	

**Post treatment**							

**1**	281.1	30.3	9.0	42.6	9.2	77	
**2**	292.6	47.9	8.5	87.4	8.4	45	
**3**	305.9	40.1	11.8	45.6	4.8	38	
**4**	357.9	66.7	14.5	75.0	4.9	34	
**5**	40.8	46.1	8.6	31.4	11.4	55	
**6**	99.1	18.0	4.5	78.3	6.8	40	
**7**	219.6	38.7	2.2	40.4	4.5	54	
**8**	411.9	53.3	3.9	60.5	4.9	49	
**9**	72.4	68.4	31.2	30.7	4.3	25	
**10**	92.3	53.5	12.8	22.8	6.7	63	
**11**	75.9	77.6	14.6	44.1	3.9	35	
**12**	184.0	47.4	8.9	33.8	3.4	22	
13^***^	131.2	46.1	9.9	60.7	6.0	26	
**14**	359.2	33.7	13.7	53.7	8.3	36	
**m ± SD**	**209 ± 126**	**48 ± 16**	**11 ± 7.0**	**51 ± 20**	**6.3 ± 2.3**	**42.8± 15.5**	

* Parameters with significant statistical differences between pretreatment and during treatment (p < 0.05)

*** *PET/CT, Diffusion-weighted MR images and TICs are given inFigures 4 and 2*.

F_p_ = blood flow; v_e_ = extracellular, extravascular fraction; v_p_ = fraction of vascular space; PS = transfer constant, L = larynx; OC = oral cavity
